# On the generalized Snell–Descartes laws, shock waves, water wakes, and Cherenkov radiation

**DOI:** 10.1515/nanoph-2024-0447

**Published:** 2025-01-17

**Authors:** Patrice Genevet, Nate Wright, Jayden Johnson, Aloke Jana, Emil Marinov, Loubnan Abou-Hamdan

**Affiliations:** Department of Physics, 3557 Colorado School of Mines, 1523 Illinois St., Golden, CO 80401, USA; Université Cote d’Azur, CNRS, CRHEA, Rue Bernard Gregory, Sophia Antipolis, 06560, Valbonne, France

**Keywords:** metasurfaces, metamaterials, Snell-Descartes laws

## Abstract

The modification of light’s trajectory after refracting through a boundary separating two media is a ubiquitous phenomenon in nature. The laws governing such refraction/reflection, known today as the Snell–Descartes laws of reflection and refraction, were established over four centuries ago and have since become foundational to the field of classical optics. Presently, with the advent of nano-photonic technology, a generalized version of these laws has been developed and implemented, vastly broadening the breadth of light manipulation methods. Despite their popularity, however, a simple and accessible derivation of the Snell–Descartes laws is still lacking, and their generalization is still largely missing from the physics curricula. Here, we use simple analogies between light’s refraction and reflection and other *a priori* unrelated radiating wave systems, namely, shock waves, water wakes, and Cherenkov radiation to derive both the classical and generalized Snell–Descartes laws, relying solely on simple and intuitive arguments. The basis of the derivation considers the excitation of a surface perturbation, induced by light incident at an angle on a boundary, that propagates at a velocity exceeding the phase velocity of light in the medium. The perturbation thereafter acts as a radiative source that reflects and refracts light away from the interface, at angles satisfying the classical Huygens interference condition. These derivations are meant to be accessible to a broad range of readers, including students of all levels, middle/high school teachers, and beyond.

## 1 Introduction

Snell’s Law, a fundamental principle in optics, was formulated by the Dutch mathematician and astronomer Willebrord Snellius (Willebrord Snel van Royen) in 1621. This law describes the relationship between the angles of incidence and reflection (respectively *θ*_*i*_ and *θ*_*r*_ in medium 1) and refraction (*θ*_*t*_ in medium 2) when light passes through the boundary between two different media of indices of refraction *n*_1_ and *n*_2_, respectively. The law states that the ratio of the sines of these angles is equal to the ratio of light’s phase velocities (or, equivalently, the ratio of the refractive indices *n*_1_ and *n*_2_) in the two media. The laws of reflection and refraction are formally expressed as follows

(1) $$\theta_{r}=\theta_{i},$$

(2) $$n_{1}\sin\theta_{i}=n_{2}\sin\theta_{t}.$$

Although the law given in Eq. (2) bears Snell’s name, it had been previously discovered by others (albeit in a slightly different form) such as the Persian scientist Ibn Sahl, a profound scholar during the Islamic Golden Age [1], and later by René Descartes during the 17th century, and as such has become known as the Snell–Descartes law of refraction. Equation (2) is crucial for the understanding of various optical phenomena, such as the bending of light in lenses and the behavior of light in different materials, and as such has become a foundational principle for much of modern optics.

**Figure 1:**
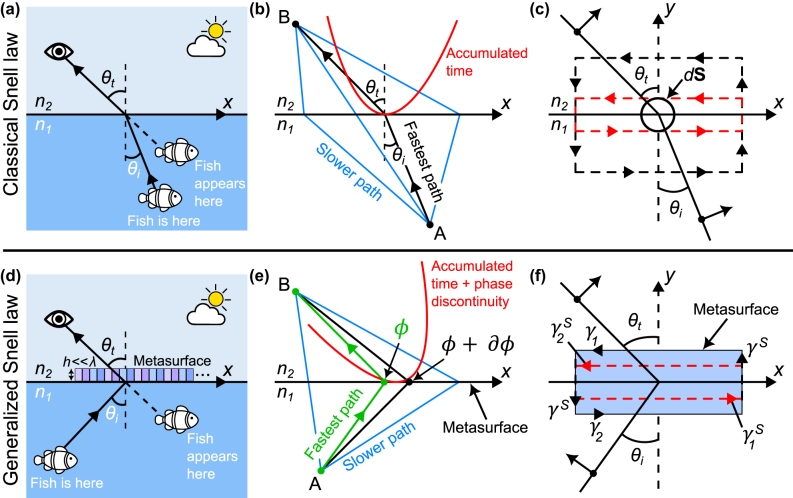
An overview of the derivation methods of the Snell-Descartes laws. (a) Light refracting across a boundary separating two different materials of indices of refraction *n*_1_ (water here) and *n*_2_ (air here) bends according to the Snell–Descartes law of refraction. As a result, an observer located above the water (denoted as medium 2 here) will find it difficult to determine the exact position of a fish in the water medium (denoted as medium 1 here). (b) The bending angles of light at the interface can be mathematically calculated using Fermat’s principle of least optical path or least accumulated phase. This approach relies on using the relatively involved mathematical concept of minimization of the integral of the phase along the path of light with respect to an infinitesimal variation of the path. (c) A second method proposed to calculate the bending angles relies on solving the boundary conditions of Maxwell’s equations. These are advanced mathematical (local) conditions, which subtly assume the notion of translational invariance along the surface. (d) The light bending angles, both in reflection and in refraction, can be arbitrarily controlled by introducing abrupt phase shifts at the interface over the scale of the actual wavelength of light, which certainly results in further confusion for the observer looking at a fish in the water. (e) Similarly to panel (b), the generalized laws can be derived by using the principle of least accumulated phase, but taking into account that infinitesimally separated paths would acquire additional but slightly different abrupt phase delays caused by the presence of the metasurface elements. (f) Finally, the most mathematically involved theoretical derivation relies on solving the generalized sheet boundary conditions, taking into consideration the discontinuous fields across a boundary. This panel clearly illustrates the conceptual difficulty of deriving either the “classical” Snell–Descartes laws or the generalized versions of the laws of reflection and refraction.

The effect of refraction is routinely experienced for example by fishermen observing objects submerged in water through the air–water interface. Figure 1(a) illustrates the difference between the perceived and the actual ray trajectories of light from a fish in water. Light reflecting off the skin of the fish propagates towards an observer above the water at an angle *θ*_1_ with the normal to the interface. Upon refraction at the interface, the light rays refract at an angle *θ*_2_ following Eq. (2). Interestingly, because the light rays carrying the fish’s image bend at the interface, the fish appears to be closer to the interface than it actually is [see Figure 1(a)]. Despite being a fundamental principle in physics, a derivation of the Snell–Descartes law, governing the phenomenon illustrated above, based on physically accessible observables is still largely missing. Even in its simplest form, the derivation of Snell’s law requires some mathematical manipulations that may be too advanced for beginner students.

In this manuscript, we provide a simple derivation of the Snell–Descartes laws, which relies on simple concepts that are accessible to anyone with a basic understanding of linear algebra. We derive the relations governing the angles of reflection and refraction of light by introducing the notion of super-sonic and super-luminal perturbations and exploiting the intuitive analogy with radiating perturbations, such as the wakes emerging behind a boat and the sonic boom. Our interpretation of the surface perturbation caused by light incident upon a surface is further extended to the case of interfaces patterned with an array of phase-delaying nano-structures (a metasurface). This last derivation is shown to be in perfect agreement with the pioneering work on the “generalized laws of reflection and refraction” [2]. The derivation methods presented herein are intended to provide all readers with an accessible and intuitive understanding of light refraction at interfaces in general, thus addressing both the “classical” light refraction problem, as well as the “generalized” light refraction problem, which is a compelling contemporary development at the origin of metasurface technology [3], [4], [5], [6], [7], [8].

## 2 Overview of existing derivation methods

In general, the various methods used to derive the Snell–Descartes laws are based on the assumption that the phase velocity of light varies when traveling through different media. This may seem trivial for experienced scholars, but it is, in fact, a vital component that is deeply rooted in the wave propagation equation. The phase velocity of light traversing a non-vacuum medium is given by

(3) $$v=\frac{c}{n},$$

where *n* is the medium’s index of refraction and *c* ≈ 3 × 10^8^ m/s is the speed of light in vaccuum.

### 2.1 Derivation from Fermat’s principle

The most widely utilized approach to derive Snell’s law involves Fermat’s principle of least time, which states that light traversing between two points A and B travels along the path that takes the least amount of time [9], as schematically illustrated in Figure 1(b). This formulation might be misleading and the derivation of the Snell–Descartes laws using this method is not necessarily obvious. The proper derivation of Fermat’s principle involves the calculation of the so-called optical path length from A to B: ${OPL}_{0}=\int_{\text{A}}^{\text{B}}n\left(s\right)ds$ (the refractive index *n* is a continuous function of the position *s*), and solving for the path that minimizes OPL_0_, *i.e.*, the path that sets the variation of the optical path length equal to zero [*δ*(OPL_0_) = 0]. Note that the phase accumulated along this optical length is given by $\varphi_{\text{accum}}=\frac{2\pi }{\lambda }{\text{OPL}}_{0}$ , where *λ* is the wavelength of light in vacuum. While relatively intuitive and mathematically accessible for students learning physics at a higher level, this derivation is inappropriate for students at the introductory physics course level. Interested readers are referred to Ref. [10].

### 2.2 Derivation from Huygens’ principle

Christiaan Huygens provided a derivation of the laws of reflection and refraction in the 17th century in his *Traité de la Lumière* [11], which he began writing in Paris in the late 1,670s but was only published later in 1,690, several years after he had returned to the Netherlands. His derivation was based on the principle bearing his name, which states that every point on a wavefront serves as a source of secondary wavelets and that the new wavefront is the tangential surface to all the secondary wavelets. Using this principle, Huygens defined two triangles, one corresponding to the incident wavefront (traveling at speed *v*_1_) and the other constructed from the tangent to the wavelets of the reflected (or refracted) wavefronts (traveling at speed *v*_2_). The two triangles share a side and the triangle corresponding to the incident wavefront has a side that is equal to *v*_1_ *t*, while the triangle corresponding to the reflected (or refracted) wavefront has a side that is equal to *v*_2_ *t*, where *t* is the time taken for the wavefronts to traverse the path (which was assumed to be equal for the two wavefronts). Using trigonometric relations, Huygens was then able to derive the laws of reflection and refraction, providing a wave-theory-based explanation for the laws previously formulated by René Descartes. The reader is referred to Ref. [12] for the detailed derivation.

The laws of reflection and refraction are derived in the current manuscript using arguments that are intimately related to the above-mentioned concepts. While the geometric construction based on the Huygens principle described above is elegant and relatively simple, its extension to the derivation of the generalized Snell–Descartes laws (see subsection 2.4), however, is not straightforward.

### 2.3 Derivation from Maxwell’s equations

Another commonly adopted approach to deriving the Snell–Descartes laws uses specific boundary conditions applied to Maxwell’s equations of electromagnetic fields and the continuity of plane wave solutions propagating on both sides of the interface [see Figure 1(c)]. Utilizing these equations along with some basic trigonometry and using the fact that the speed of light in a medium is $v=1/\sqrt{\varepsilon \mu }$ , where *ɛ* and *μ* are the permittivity and permeability of the medium, respectively, one eventually arrives at the usual relations between the incident and reflected/refracted angles. While elegant, this approach is far too mathematically intensive and unintuitive to be a useful teaching tool for all student levels. The interested reader is referred to Ref. [13] for more details.

### 2.4 The generalized Snell–Descartes laws

A new degree of freedom in light manipulation has been recently achieved using nano-structured interfaces or metasurfaces. Metasurface technology has already greatly impacted the field of optics by enabling almost arbitrary light properties [see Figure 1(d)]. Arbitrary and subwavelength light modulation are engineered via the scattering response of subwavelength arrays of nano-resonators of various shapes, materials, and orientations. This field expanded rapidly after the demonstration of arbitrary reflection and refraction at interfaces designed to introduce spatially varying abrupt phase discontinuities in the form of a gradient. In this seminal work, the application of Fermat’s principle in the presence of phase-delaying nano-structures at interfaces led to the following generalization of Eqs. (1) and (2) [2]:

(4) $$n_{1}\sin\theta_{r}=n_{1}\sin\theta_{i}+\frac{\lambda }{2\pi }\frac{\partial \phi }{\partial x},$$

(5) $$n_{2}\sin\theta_{t}=n_{1}\sin\theta_{i}+\frac{\lambda }{2\pi }\frac{\partial \phi }{\partial x},$$

where *λ* is the wavelength of incident light in vacuum and *ϕ*(*x*) is the phase retardation introduced by the metasurface’s subwavelength resonators at position *x* along the interface.

The exact derivation of the generalized Snell–Descartes laws also works by taking the derivative of the total optical path length (OPL) with respect to an infinitesimal variation of the path and finding the condition at which this variation is zero. An additional key ingredient, however, that accounts for the presence of a phase gradient metasurface, must be introduced when deriving the generalized laws. Therefore, the new optical path length consists of the optical path length OPL_0_ accumulated along the path from point A to point B (see subsection 2.1) plus an additional contribution which is proportional to the spatially-varying abrupt phase shift *ϕ*(*x*). This additional abrupt phase shift corresponds to the local phase delay introduced by the metasurface’s subwavelength building blocks. The accumulated phase for light propagating from point A to point B [see Figure 1(e)] is intrinsically linked to the time needed for a light ray to travel from A to B. In contrast, the additional phase delaying term *ϕ*(*x*) at the boundary is independent of time but is added to the accumulated phase along each path. The total optical path length is thus given by: $OPL=\int_{\text{A}}^{\text{B}}n\left(s\right)ds+\frac{\lambda }{2\pi }\phi \left(x\right)$ . It is evident from the above that the additional term in Eqs. (4) and (5), $\frac{\lambda }{2\pi }\frac{\partial \phi }{\partial x}$ , naturally arises from the minimization of the accumulated phase with respect to adjacent paths, and as a result of this spatial derivative, this term accounts for the spatially-varying phase at the interface. A schematic used to support the above derivation is shown in Figure 1(e).

Note that if the phase discontinuity is constant, that is ∂*ϕ*/∂*x* = 0, then the conventional Snell–Descartes laws are recovered. When the phase gradient term is non-zero, a linear phase shift is introduced as a function of the position along the interface, which results in the bending of any incident plane wave by an amount proportional to ∂*ϕ*/∂*x*. The introduction of a phase gradient is conceptually equivalent to periodically repeating a phase ramp ranging from 0 − 2*π* over a period Λ. Replacing ∂*ϕ*/∂*x* by 2*π*/Λ thus transforms Eq. (4) into the well-known first-order blazed grating diffraction equation [14], [15].

Another derivation, which requires advanced mathematics, is solving the boundary conditions in the presence of surface susceptibility tensors in the space domain. This derivation uses the so-called generalized sheet transition conditions [16]. A schematic describing this derivation methodology is presented in Figure 1(f). The coefficients of these susceptibility tensors are related to the incident, reflected, and transmitted fields around the structure in a manner that satisfies the generalized sheet transition conditions [17]. The generalized sheet transition conditions calculation has also been extended to non-planar and conformable metasurfaces [18], [19]. This synthesis method is extremely powerful and can treat any arbitrary electromagnetic transformation. However, it requires a higher level of technicality and is plagued by issues associated with solving inverse underdetermined problems, for which the system of polynomial equations has fewer equations than the number of the tensor coefficients. In addition, these theoretical calculations often lead to ideal synthesized values that might be impossible to realize in a physical system.

All existing derivations of the generalized Snell–Descartes laws thus require a mathematical background that exceeds the level of most students and scholars. To make these laws more accessible, we formulate these laws using a different approach that is likely to trigger the interest of non-experts and may also highlight the light manipulation potential of metasurfaces for various types of optical devices and systems, such as flat lenses [20], [21], laser wavefront shaping [22], polarimetry [23], holography [24], augmented reality/virtual reality [25], and light detection and ranging [26].

## 3 Proposed derivation of the Snell–Descartes laws

In this section, we first provide a different approach to derive the classical laws of reflection and refraction using first principles based on physical arguments. To do so, we must first introduce the notion of a surface perturbation. The physical representation of a surface perturbation is more readily comprehensible by making an analogy with familiar wave phenomena, such as water waves breaking on a shore. In what follows we calculate the speed of breaking waves along the shore, and, in analogy with a sonic boom, we introduce the notion of an excitation exceeding the speed of the wave. We further discuss how such a “super-luminal” perturbation radiates light. Simple geometric formulae relating the surface perturbation to the radiated light are proposed and are used to calculate the reflected and refracted angles of light, which are found to be in perfect agreement with the Snell–Descartes relations mentioned previously.

We underscore here that despite the apparent super-luminal speed, this new analysis remains physically valid since the super-luminal perturbation considered in the following carries no information, and thus remains harmonious with the fundamental principle that information cannot be transferred faster than the speed of light. This is due to the fact that optically-encoded information can only be carried by the incident and reflected (or refracted) beams, which travel at the phase velocity in the medium.

### 3.1 Definition of the fast running perturbation: the notion of the running wave of polarization

**Figure 2:**
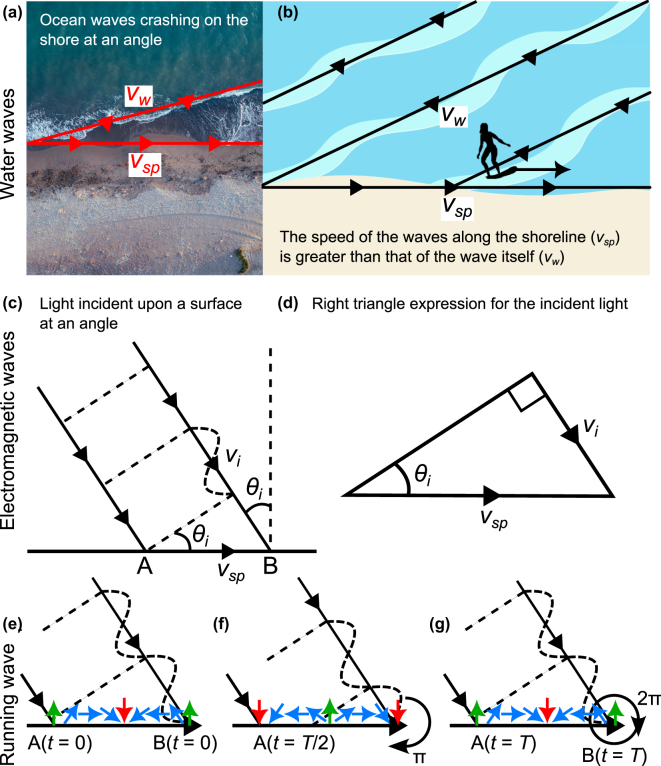
An anology between ocean waves crashing on a shore and a plane electromagnetic wave impinging on an interface. (a) An observer sitting on the shore, watching the ocean waves approach the coastline at an angle witnesses a rather interesting phenomenon: the speed of the crashing point of the wave along the shoreline, denoted as *v*_*sp*_, always exceeds the speed of the incoming waves itself, denoted by *v*_*w*_. (b) This effect is well-known to surfers who travel the globe in search of waves with relatively large breaking peel angles [27]. (c) By analogy, the light impinging at an interface interacts with the surface material, creating a surface perturbation wave, or a running wave of polarization, that propagates at a faster speed than the incident light itself. (d) A simple right triangle expression is used to express the surface perturbation speed as a function of the incident angle. (e)–(g) Three more technical visualizations of the above-mentioned interaction process. In panel (e), light polarizes the medium, forming a line of phased dipoles that linearly increase in phase from 0 → 2*π* across two consecutive equi-phase points on the interface. Note that this distance is simply given by Δ*x* = *λ*_1_/sin*θ*_*i*_. (f) After half of a period *t* = *T*/2 of propagation of the incident light, the dipoles have all been shifted along the interface over the distance Δ*x*/2. For example, the dipole initially positioned at A at *T* = 0 represented by the green phase value, is now positioned at Δ*x*/2. (g) After a full period of propagation, the incident light would have displaced all of the dipoles along the line over the entire Δ*x* distance. It took a period *T* for all dipoles forming the surface perturbation wave to cover a distance Δ*x*, as a result, the speed of the surface perturbation is *v*_*sp*_ = *v*_*i*_/sin*θ*_*i*_ = *c*/*n*_1_ sin*θ*_*i*_.

The simplest way to think of the notion of a running wave of polarization is to draw analogies to ocean waves crashing on a shore [Figure 2(a)]. If the waves propagate at a speed *v*_*w*_ and hit the shore at an angle *θ*_*i*_, then the speed of the crashing waves along the shore *v*_*sp*_ is given by

(6) $$v_{sp}=\frac{v_{w}}{\sin\theta_{i}}.$$

This calculation also roughly approximates the speed of a surfer riding the breaking waves towards the shore, as shown in Figure 2(b). Research on surfing has revealed that for a wave to be surfable, the wave has to break gradually along the wave crest and not all at once. This breaking point is called the “peel”, and the velocity at which this happens is thus called the “peel rate” *v*_*sp*_ of the wave, which is related to the speed of the surfer. In other words, it is possible to relate the velocity of the peel and the velocity of the wave *v*_*w*_ via the peel angle (*θ*_*i*_) forming between the two vectors. This is certainly the most important parameter as it defines whether a wave is surfable or not. If this angle is too small, the velocity of the breaking point becomes extremely large, which essentially means that the wave breaks all at once along the crest wave. Waves suitable for surfing require a relatively large angle between the wave and the peel [27].

It can be seen immediately from Eq. (6) that the wave speed along the shore exceeds the speed of the water waves for any angle *θ*_*i*_ ≠ 90°. Concordantly, an observer sitting on the shore would see the speed of the crashing waves along the shoreline becoming infinitely larger as the incident angle *θ*_*i*_ → 0. It is in this sense that a surface perturbation can exceed the speed of the wave itself.

Similarly to ocean waves hitting the shore at an angle, consider now a linearly polarized plane light wave impinging on an interface between two media at an angle *θ*_*i*_ [Figure 2(c)]. A plane wave is a wave consisting of multiple transverse plane wavefronts that are repeated after the wave propagates a distance that is equivalent to its wavelength. The transverse plane wavefronts are planes in which the wave maintains the same oscillation properties (*i.e.*, the wave has constant phase and amplitude). The linear polarization corresponds to a given orientation of the incident electric field. The phase velocity of the incident light is *v*_*i*_ = *c*/*n*_1_, where *n*_1_ is the index of refraction of the medium from which the wave is incident. The speed at which the running wavefront “crashes” on the interface is obtained from the right triangle representation of Figure 2(d).

In more technical terms, when “crashing” on the interface the light wave continually polarizes the surface with a phase value that linearly varies between 0 − 2*π* along two consecutive equi-phase fronts reaching the surface denoted by points A and B in Figure 2(c). The speed *v*_*sp*_ can thus be calculated from geometric arguments, considering that the polarization wave propagates the distance separating A and B, Δ*x* during exactly one period of oscillation *T*. Hence, the speed of the surface perturbation is given by

(7) $$v_{sp}=\frac{\Delta x}{T}=\frac{\lambda_{i}}{T\sin\theta_{i}}=\frac{v_{i}}{\sin\theta_{i}}=\frac{c}{n_{1}\sin\theta_{i}},$$

where *λ*_*i*_ = *λ*/*n*_1_ is the wavelength in the incidence medium. As light propagates forward, the local value of the phase of light impinging at a given location on the interface changes at a speed *v*_*sp*_. Equation (7) thus defines the moving speed of the running wave of polarization induced by the incident light. In the following, we will treat this running wave of polarization as an optical perturbation.

The expression in Eq. (7) indicates that the speed of this perturbation along the surface propagates at a speed exceeding the phase velocity of light *v*_1_ in the medium of incidence. To illustrate the relationship between the phase of the incident light with the phase of the surface perturbation, that is, the light propagation along the surface, we show in Figure 2(e)–(g) the evolution of the surface polarization (represented by arrows) as a function of time during one period of propagation *T*. In Figure 2(e) we define the initial phase of the surface polarization to be zero, as denoted by the upright arrow at *t* = 0 at the location A on the surface. After half a period [Figure 2(f)], the incident wavefront had propagated forward by a distance of half a wavelength, and as a result, the phase of the excited polarization wave at A has changed by an amount of *π*, equivalent to half a period of propagation. After one period (*t* = *T*) of propagation [Figure 2(g)], the phase at A would have rotated by an amount of 2*π*, equivalent to one period of propagation. The distance Δ*x* propagated by the polarization wave during the period of time *T* is used to retrieve the super-luminal speed *v*_*sp*_ of Eq. (7).

### 3.2 The Snell law analogy: the sonic boom, wakes, and Cherenkov radiation

In this subsection, we show how a super-luminal (*v*_*sp*_ > *c*/*n*) excitation results in the radiation of reflected and refracted light beams. According to Huygens’ principle, wave propagation is intuitively understood by considering that every point on a wavefront is a source of wavelets spreading out in the forward direction at the same speed as the wave itself (*v*_*i*_). All the spherical waves propagating forward interfere, forming the new propagated wavefront at the plane tangent to all of the wavelets. The Huygens principle applies to all types of waves, including water waves, sound waves, and light waves (electromagnetic waves). In the following, we will make use of Huygens’s argument and analogies with other wave radiative systems to explain how our optical perturbation reflects and refracts light in agreement with the laws of reflection and refraction.

**Figure 3:**
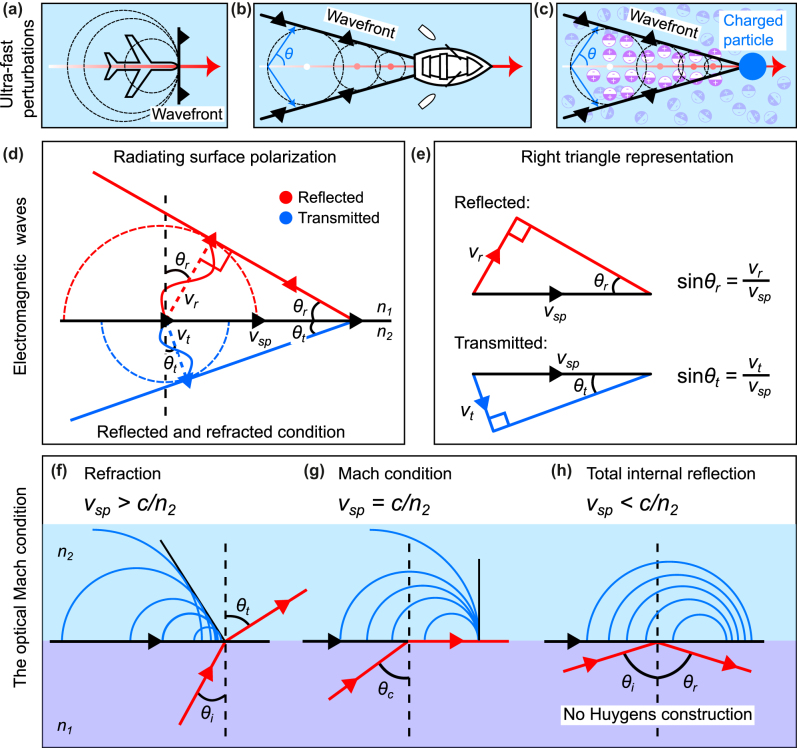
Ultra-fast perturbations, electromagnetic waves, and the optical Mach condition. (a)–(c) Examples of conical wave radiating systems. Waves are formed when an object travels faster than the speed of the wave in the respective medium. This behavior is general and can be observed in various systems including sound waves, water waves, and electromagnetic waves. (a) The so-called Mach condition corresponds to the case where pressure waves accumulate at the forefront of an aircraft flying at the exact speed of sound. (b) A boat propelled in the forward direction creates wakes that emerge sideways at a given angle from the boat’s trajectory. The boat behaves as a moving line of perturbation at the surface of the water, displacing water and creating circular ripples centered at the boat’s position. When the boat is moving faster than the speed of the surface water waves, the ripples will interfere creating wakes behind the boat. (c) Cherenkov radiation, often observed in underwater nuclear reactors, occurs when charged particles traverse a medium at velocities exceeding the phase velocity of light in the considered medium. (d) In analogy with the radiating systems discussed in panels (a)–(c), the reflection and refraction of light are related to induced coupled charges traveling parallel to the interface at a speed exceeding the speed of light in the considered media 1 and 2 of indices of refraction *n*_1_ and *n*_2_, respectively. (e) Simple trigonometric constructions linking the speed of the surface perturbation *v*_*sp*_ to the speed of light in the two media. (f) Refraction from medium 1 to 2 occurs when the surface perturbation propagates faster than the speed of light in medium 2, *i.e.*, *v*_*sp*_ > *c*/*n*_2_. (g) The critical angle *θ*_*c*_ = sin^−1^(*n*_2_/*n*_1_) is the optical analog of the Mach condition in acoustics. In this case, light propagates along the interface. (h) When the surface perturbation speed is slower than the light in medium 2 (total internal reflection), the emitted waves cannot interfere constructively, a wavefront tangent to all the secondary wavefronts is unattainable in this case. The sources are emitted within each other, and no Huygens construction is possible. The light is only reflected in medium 1.

We propose to start here again with more familiar wave phenomena. Figure 3(a) and (b) depict everyday examples of radiating wave systems, namely, sonic waves produced by an airplane and water wakes produced by the motion of a boat. In today’s world, the thunderous noise produced by an aircraft breaking the sound barrier, *i.e.*, traveling faster than the speed of sound, is rather commonplace. This noise is caused by compressed moving sound waves, called shock waves. The aircraft causes a line of super-sonic perturbation along its trajectory, from which sound waves are emitted. The direction where these waves merge in phase generates a pressure wave responsible for the sonic boom [see the tangent of all circles forming the wavefront in Figure 3(a)]. A very similar effect is observed with water waves when a boat navigates along a certain trajectory, creating a line of perturbation at the surface of the water from which spherical waves are generated. The direction of the wakes forming behind the boat can also be obtained by considering the direction of constructive interference of all radiating sources [see again the tangent to all spherical waves in Figure 3(b)].

Another example of an ultra-fast perturbation that is known to emit radiation, although less known to the general public, is the Cherenkov radiation effect. This effect is named after Pavel Alekseyevich Cherenkov, a Soviet scientist who won the 1958 Nobel Prize in Physics for his experimental observation of bluish-colored light radiating from nuclear water tanks [28]. Blue light is produced by charged particles generated during a nuclear reaction process when they pass through an optically transparent medium at speeds greater than the phase velocity of light in that medium. Again, the charged particles can be considered as a perturbation propagating in an optical medium. As the charged particle propagates, it locally polarizes the material, leaving behind a line of perturbed polarization along its trajectory. The relaxation of the medium results in the emission of spherical waves centered along the trajectory of the charged particle, as shown in Figure 3(c). The constructive interference of all of these radiated waves creates a cone of light propagating at an angle from the particle’s trajectory.

Although it may seem rather unlikely at first glance, all of the above-mentioned physical processes of wakes, sonic booms, and even Cherenkov radiation, are akin to the reflection and refraction of light. As it has been previously shown [29], such radiative mechanisms are indeed somehow identical and are naturally occurring as a consequence of the Huygens principle.

To calculate the refraction and reflection angles, we now consider that light can be radiated in both the medium of incidence and the medium of transmission (media 1 and 2 with indices *n*_1_ and *n*_2_, respectively), as a consequence of the optical perturbation, *i.e.*, the running wave of polarization propagating at speed *v*_*sp*_. The schematic in Figure 3(d) depicts both “optical wake” signals in media 1 and 2, shown in red and blue colors, respectively. The construction used to calculate the respective angles considers that, during a given period of time, for example, one period of oscillation *t* = *T*, the emitted field in both respective media have propagated a distance *v*_*r*_ *T* = *λ*_1_ and *v*_*t*_ *T* = *λ*_2_, where *v*_*r*_ and *v*_*t*_ are the speeds of the reflected and transmitted waves in media 1 and 2, respectively. Solving for the trigonometric relationship linking the distances of the running wave of polarization, that is, Δ*x* = *v*_*sp*_ *T* with the distances propagated by light in media 1 and 2 during a time *T* yields [see Figure 3(e)]:

(8) $$\sin\theta_{r,t}=\frac{\lambda_{1,2}}{\Delta x}=\frac{v_{r,t}}{v_{sp}}.$$

Note that the velocity of the running wave of polarization *v*_*sp*_ depends only on the incident angle *θ*_*i*_ [see Eq. (7)]. Notice also that *v*_*sp*_ is identical for both reflected and refracted right triangles. We thus obtain the following relationships:

(9) $$\frac{v_{i}}{\sin\theta_{i}}=\frac{v_{r}}{\sin\theta_{r}}\;and\;\frac{v_{i}}{\sin\theta_{i}}=\frac{v_{t}}{\sin\theta_{t}}.$$

Using the expression for the phase velocity in Eq. (3), we can rewrite the above expressions in terms of *c*, *n*, and *θ*_*i*,*r*,*t*_. This elegantly leads to the expected laws of reflection and refraction. *i.e.*, Eqs. (1) and (2).

It should be noted here that Eq. (1) is always true, however, Eq. (2) is only valid for *θ*_*i*_ ≤ *θ*_*c*_, where *θ*_*c*_ is the critical angle of incidence (also referred to as the angle of total internal reflection), satisfying the condition sin*θ*_*c*_ = *n*_2_/*n*_1_. Within the physical picture considered here, this condition exactly corresponds to the Mach condition, that is, the condition at which the speed of the perturbation *v*_*sp*_ exactly matches the velocity of the wave in the medium of transmission, *v*_*sp*_ = *v*_*t*_. This specific case is depicted in Figure 3(g). In contrast, when the surface perturbation wave created by the incident light propagates slower than the phase velocity of light through the transmission medium on the other side, refraction vanishes [see Figure 3(h)] since it is impossible to construct a line tangent (a wavefront) to all of the spherical waves previously emitted by the surface, as depicted in Figure 3(f). The destructive interference, in this case, imposes an exponentially decaying intensity away from the surface, giving rise to an evanescent electric field confined to the interface. Note that a similar analysis might be considered in the framework associated with the boundary layer separation in hydrodynamics for the study of perturbations at the interface between two liquids of different viscosity. However, such a mechanism involves a non-linear regime that extends beyond the simple boundary-layer model and thus beyond the scope of the current study [30], [31].

## 4 Derivation of the generalized Snell–Descartes laws using the surface perturbation approach

**Figure 4:**
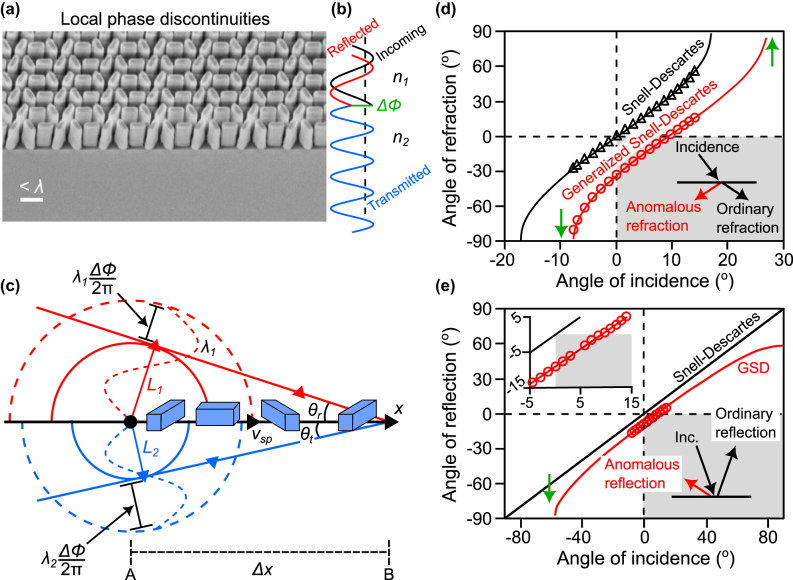
Phase-delaying nano-structuration for anomolous reflection and refraction. (a) Scanning-electron microscope image showing a typical example of spatially rotated nano-structures that are utilized to introduce a spatially varying phase delay. (b) Light impinging on an interface between two media of indices *n*_1_ and *n*_2_ is delayed by introducing abrupt phase changes at the interface, denoted as Δ*ϕ*, over the scale of the wavelength. (c) The presence of nano-structures patterned at a subwavelength distance along the interface between two media introduces a spatially distributed phase retardation Δ*ϕ* on the surface perturbation, thus modifying the speed of the surface perturbation. (d) Angle of refraction versus angle of incidence for the ordinary (black curve and triangles) and anomalous refraction (red curve and circles). The curves (solid lines) are theoretical calculations from the generalized Snell’s law of refraction [Eq. (5)], and the symbols are experimental data extracted from refraction measurements as a function of the angle of incidence. The shaded region represents “negative” refraction. The green arrows indicate the modified critical angles for total internal reflection. (e) Angle of reflection versus angle of incidence for the ordinary (black curve) and anomalous (red curve and circles) reflection. The curves are theoretical calculations from the generalized Snell–Descartes (GSD) law of reflection [Eq. (4)] and the symbols are experimental data extracted from reflection measurements as a function of the angle of incidence. The shaded region represents “negative” reflection. The green arrow indicates the critical angle of incidence above which the anomalously reflected beam becomes evanescent. (Panels (d) and (e) were reproduced from Ref. [2]).

In their traditional form, the usual Snell–Descartes laws only apply to smooth, flat surfaces. In the absence of surface structuration, the interface is translationally invariant along the in-plane direction, and as a result of the continuity of the electromagnetic field, the in-plane component of the fields is identical in both media across the boundary. This somewhat simplifies the derivation of the Snell–Descartes laws. In 2011, researchers proposed a generalization of the Snell–Descartes laws [see Eqs. (4) and (5)], extending the concepts of reflection and refraction to more complex scenarios, including surfaces with phase gradient-inducing micro/nano-structures or metasurfaces [see Figure 4(a) and (b)] that can manipulate light in unconventional ways. These advanced versions of the laws account for additional factors like surface shape and subwavelength-scale engineered materials, allowing for precise control over light’s properties. This has opened exciting new possibilities in technologies like advanced flat lenses, holography, and optical communication, pushing the boundaries of light manipulation to various innovative applications [6], [32], [33], [34], [35]. However, as previously mentioned, the derivation of the generalized laws may be challenging for non-experts. In the following, we apply our approach of a fast-moving perturbation with the addition of a phase discontinuity at the interface, to derive the generalized Snell–Descartes laws. It is worth mentioning here that the following formulation is only applicable in the context of nano-photonics, where the optical response of subwavelength-sized structuration can be replaced by an overall effective interfacial response.

In contrast to the previous section, we now introduce a spatially-varying phase retardation (known as a phase shift in electromagnetism) at the interface between the two media 1 and 2. Figure 4(c) illustrates the effect of adding a metasurface that introduces a linear phase gradient ∂*ϕ*/∂*x* in the *x*-direction along the interface. The local phase retardation results in a local delay of the emission, which between two points of the surface, separated by a distance Δ*x*, is given by Δ*ϕ*(*x*).

### 4.1 Derivation using the wavefront propagation distance

The first derivation of the generalized Snell–Descartes laws entails the decomposition of the emission in media 1 and 2 following two consecutive processes. (i) We first consider that the incident light creates the surface perturbation of speed *v*_*sp*_ in agreement with the previously discussed excitation mechanism. (ii) This surface perturbation radiates spherical waves in the considered medium, however, due to the presence of the local phase elements, *i.e.*, the patterned nano-resonators [Figure 4(c), blue rods], the phase at which each spherical wave is emitted is now delayed by an amount equal to the local phase delay Δ*ϕ*(*x*) induced by the nano-resonator.

Considering this problem over a period of oscillation *t* = *T*, the surface polarization excited by the incident light, which propagates at the speed *v*_*sp*_, would have moved along the interface a distance

(10) $$\Delta x=v_{sp}T=\frac{\lambda }{n_{1}\sin\theta_{i}}.$$

This Δ*x* is the same as the one introduced in the previous section. Using the expression for the phase gradient, the additional phase difference induced by the metasurface between two points separated by Δ*x* is expressed as $\Delta \phi =\frac{\partial \phi }{\partial x}\Delta x$ . It should be noted here that both the phase gradient and Δ*x* are algebraic values and could be negative depending on the system under consideration. Due to this delay, the wavefront emitted by a point A excited a period *T* ahead of time and positioned at a negative distance Δ*x* (considering the origin is at a point B) would have propagated in media 1 and 2 a distance *L*_1_ and *L*_2_, respectively [see Figure 4(c)], which are given by

(11) $$L_{1,2}=\lambda_{1,2}+\lambda_{1,2}\frac{\Delta \phi }{2\pi },$$

where $\lambda_{1,2}\frac{\Delta \phi }{2\pi }=\frac{cT}{n_{1,2}}\frac{\Delta \phi }{2\pi }$ represent the equivalent of light propagation in media 1 and 2 associated with the amount of phase delay at the position Δ*x*. From the schematic in Figure 4(c), we obtain the following trigonometric relations:

(12) $$\sin\theta_{r}=\frac{L_{1}}{\Delta x}\;and\;\sin\theta_{t}=\frac{L_{2}}{\Delta x}.$$

Using Eq. (10), $\Delta \phi =\frac{\partial \phi }{\partial x}\Delta x$ , and the fact that Δ*x* = *λ*/*n*_1_ sin*θ*_*i*_, in the above relations yields the generalized laws of reflection and refraction of Eqs. (4) and (5).

### 4.2 Derivation using the modified emission/excitation velocities

We note here that the tedious and non-intuitive consideration of an overall effective distance associated with the negative phase delay −|Δ*ϕ*| at the position A with respect to B can in principle be avoided using Eq. (8) instead.1The term effective distance corresponds to the equivalent distance that light traverses in free-space to produce a phase-delayed wavefront Δ*ϕ* during one period of oscillation *T*.
Equation (8) is the generic expression for calculating the radiative angle of waves excited by fast perturbations. Here, there are two different ways of expressing the effect of the phase-delaying elements: Either we consider that (i) the phase response of the interface modifies the speed of the running wave of polarization, *i.e.*, we replace *v*_*sp*_ by $v_{sp}^{\prime }\left(\Delta \phi \right)$ , and keep the radiation speed in media 1 and 2 equal to the phase velocity in media 1 and 2 (*v*_*r*,*t*_ = *c*/*n*_1,2_). Or we assume that (ii) the incident wave excites the previously mentioned running wave of polarization at the speed *v*_*sp*_ = *c*/*n*_1_ sin*θ*_*i*_, but that the speeds *v*_*r*,*t*_ of the reflected and transmitted waves are replaced by the speeds $v_{r,t}^{\prime }$ , which are modified by the introduction of phase-delaying elements at the interface.

Rewriting the general expression in Eq. (8) with the modified speeds, we obtain:

(13) $$\sin\theta_{r,t}=\frac{v_{r,t}^{\prime }\left(\Delta \phi \right)}{v_{sp}}=\frac{v_{r,t}}{v_{sp}^{\prime }\left(\Delta \phi \right)},$$

with

(14) $$v_{r,t}^{\prime }\left(\Delta \phi \right)=v_{1,2}\left(1+\frac{\Delta \phi }{2\pi }\right),$$

(15) $$v_{sp}^{\prime }\left(\Delta \phi \right)=\frac{v_{sp}}{\left(1+\Delta \phi /2\pi \right)},\;$$

where we added the speeds in “series” for the modified radiation speed terms, or in “parallel” for the modified surface wave excitation speed. Utilizing the expression for *v*_*sp*_ given in Eq. (7), both approaches illustrated in the above equations lead to the generalized laws of reflection and refraction, where the difference in the sines of the angles of the reflected and incident waves weighted by their indices of refraction is now accompanied by an additional term proportional to the spatial phase gradient multiplied by the inverse of the light wave’s wavenumber.

A remarkable outcome of the generalized Snell–Descartes laws is that given a suitable phase gradient along the interface, an arbitrary refraction angle can be achieved, as shown in Figure 4(d). The introduction of the phase gradient term can be considered as an additional transverse momentum that asymmetrically changes the refraction angle as a function of the incident angle. Light incident from symmetrically opposite directions (±*θ*_*i*_) would now refract at two different angles. This behavior is a consequence of the algebraic value of the phase gradient effective velocity term that modifies the phase velocity [see Eq. (14)], which also results in two different critical angles at which refraction vanishes, leading to total internal reflection.

According to the discussion in subsection 3.2 and Figure 3(f)–(h), the addition of a phase gradient now asymmetrically changes the emission speed, as well as the Mach condition, whether light impinges at positive or negative incident angles. This phase gradient term also strongly modifies conventional specular reflection, as illustrated in Figure 4(e). Incidentally, Eq. (4) predicts a critical angle at which reflection vanishes, that is, above which the speed of the surface perturbation is also too slow to satisfy the Huygens’ construction in the reflection medium. As a result, the reflected beam only evanescently couples to the interface.

## 5 Conclusion

In summary, we have provided a simple derivation of the classical and generalized Snell–Descartes laws using the concept of the super-luminal phase velocity of light and relying on relatively simple arguments based on several analogies with known examples from everyday life. We illustrated how light incident upon an interface separating two media can generate a fast propagating surface perturbation, formally referred to as a running wave of polarization. The speed of this surface perturbation was calculated using simple trigonometric arguments and using familiar examples we illustrated how such a fast perturbation could radiate waves, just as an aircraft traveling faster than the speed of sound leads to the formation of shock waves. It was shown that the reflected and refracted light at the interface are emissions of light generated from a super-luminal perturbation and their respective angles were calculated via simple trigonometric relations. The methodology discussed above was also extended to the derivation of the generalized Snell–Descartes laws, in which a phase gradient is present at the interface. This phase gradient is in practice realized by patterning the surface with pre-designed nano-structures to form a metasurface.

It should be emphasized here again that when light is incident upon an interface at an angle that is close to normal incidence, *i.e.*, *θ*_*i*_ → 0, the surface perturbation speed *v*_*sp*_ → ∞. This may sound unphysical and is certainly unintuitive, and potentially conflicting with the limitations imposed by the theory of relativity. However, it is necessary to explain that this is indeed non-conflicting with other physical interpretations: the surface wave excitation mechanism cannot transfer energy at super-luminal speed, simply because the transfer of phase is performed at different points of the interface by different parts of the wavefront. Two different sections of the surface perturbation, spatially separated by a given amount along the interface, are excited by two different incident photons.

We hope that this method will prove useful for those interested in providing a more intuitive and potentially more comprehensible notion of light propagation, reflection, and refraction, to students.2This derivation method of the Snell-Descartes laws was given to high school students with no prior knowledge in the field of optics at the Golden High School in Colorado (USA). Our approach consisted of dividing the lesson into two halves. During the first half, we presented the usual derivations of Snell’s Law, based on mathematical tools that were certainly beyond their grasp. In the second part of the lesson, we showed how Snell’s laws can be easily obtained using our trigonometric method. For the most part, the second half of the lesson was dedicated to explaining the concepts of wakes and surface perturbations, and we found that these concepts were quite comprehensible to the students. The lesson also included a series of hands-on demonstrations to complement the lecture and to keep students engaged. Gauging the students’ understanding of Snell’s law, mathematically as well as conceptually, before and after the class, we found that they were qualitatively receptive to the lecture and most of the groups were capable and excited to perform the derivation themselves. Teaching slides can be provided for teachers/people interested in using this methodology.

